# Marine Microbial Food Web Networks During Phytoplankton Bloom and Non-bloom Periods: Warming Favors Smaller Organism Interactions and Intensifies Trophic Cascade

**DOI:** 10.3389/fmicb.2020.502336

**Published:** 2020-10-23

**Authors:** Thomas Trombetta, Francesca Vidussi, Cécile Roques, Marco Scotti, Behzad Mostajir

**Affiliations:** ^1^Marine Biodiversity, Exploitation and Conservation, Centre National de la Recherche Scientifique, Institut Français de Recherche pour l’Exploitation de la Mer, Institut de Recherche pour le Développement, University of Montpellier, Montpellier, France; ^2^GEOMAR Helmholtz Centre for Ocean Research Kiel, Kiel, Germany

**Keywords:** microorganism interactions, correlation networks, phytoplankton bloom, warming, microbial food web, shallow coastal zone

## Abstract

Microbial food web organisms are at the base of the functioning of pelagic ecosystems and support the whole marine food web. They are very reactive to environmental changes and their interactions are modified in response to different productive periods such as phytoplankton bloom and non-bloom as well as contrasted climatic years. To study ecological associations, identify potential interactions between microorganisms and study the structure of the microbial food web in coastal waters, a weekly monitoring was carried out in the Thau Lagoon on the French Mediterranean coast. The monitoring lasted from winter to late spring during two contrasting climatic years, a typical Mediterranean (2015) and a year with an extreme warm winter (2016). Correlation networks comprising 110 groups/taxa/species were constructed to characterize potential possible interactions between the microorganisms during bloom and non-bloom periods. Complex correlation networks during the bloom and dominated by negative intraguild correlations and positive correlations of phytoplankton with bacteria. Such pattern can be interpreted as a dominance of competition and mutualism. In contrast, correlation networks during the non-bloom period were less complex and mostly dominated by tintinnids associations with bacteria mostly referring to potential feeding on bacteria, which suggests a shift of biomass transfer from phytoplankton-dominated food webs during bloom to more bacterioplankton-based food webs during non-bloom. Inter-annual climatic conditions significantly modified the structure of microbial food webs. The warmer year favored relationships among smaller group/taxa/species at the expense of large phytoplankton and ciliates, possibly due to an intensification of the trophic cascade with a potential shift in energy circulation through microbial food web. Our study compares a typical Mediterranean spring with another mimicking the prospected intensification of global warming; if such consideration holds true, the dominance of future coastal marine ecosystems will be shifted from the highly productive herbivorous food web to the less productive microbial food web.

## Introduction

The microbial food web encompasses several microorganisms (e.g., virio-, bacterio-, phyto-, and protozooplankton) and plays a pivotal role in marine ecosystems as it controls energy as well as organic and inorganic matter transfer either to higher trophic levels or to the water-dissolved pool (e.g., dissolved organic carbon: DOC). Phytoplankton is the main primary producer of the microbial food web, and it supports a part of ecosystem productivity by providing carbon to higher trophic levels, especially during bloom periods (Cloern, 1996). Phytoplankton is also considered as the most important source of DOC in marine environments through exudation, losses by cell damage, or lysis (Dafner and Wangersky, 2002). In aquatic ecosystems, DOC is essential for the persistence and growth of bacteria. Important amounts of carbon can pass through bacteria to higher trophic levels, showing the relevance of the microbial food web to carbon circulation and ecosystem functioning (Azam et al., 1983; Mostajir et al., 2015). Besides, bacteria play a crucial role in nutrient cycling through remineralization of organic matter benefiting phytoplankton production (Fasham, 1984). Phytoplankton and bacteria can be consumed either by metazoan (herbivorous food web) or protozooplankton (microbial food web). Heterotrophic protists are the main consumers of both bacteria and phytoplankton, and they can actively transfer energy to higher trophic levels (Weisse et al., 1990; Calbet and Landry, 2004; Calbet and Saiz, 2005). Moreover, they contribute significantly to the dissolved pool through excretion as well as egestion and dissolution of fecal material (Fuhrman, 1999). In addition, viruses often play an underestimated role in carbon cycling. They regulate matter circulation through cell lysis, thus supplying energy for bacterial production. The transfer of energy through the microbial food web is then controlled by a multiple combination of interactions and associations between different microorganisms called microbial network.

Interactions between microorganisms modulate microbial food web structure and performance by influencing the amount of energy that circulates, the intensity of nutrient cycling, and transfer efficiency to higher trophic levels. Microbial food webs embed diverse relationships that include predation, cross-feeding, competition, commensalism, mutualism, and parasitism. Predator-prey interactions transfer carbon from phytoplankton or bacterial biomass to higher trophic levels (Fenchel, 1988; Calbet and Landry, 2004). In the microbial food web, this role is played by heterotrophic and mixotrophic protists such as flagellates, naked ciliates, and tintinnids, which strongly control the biomass of their resources and regulate energy transfer to higher trophic levels like planktonic crustaceans, molluscs or fish larvae (Azam et al., 1983; Weisse et al., 1990; Mostajir et al., 2015). The excretion of inorganic or organic matter by grazers also contributes to the organic matter pool, and it facilitates the growth of microorganisms such as bacteria and phytoplankton (Snyder and Hoch, 1996) in a process known as cross-feeding (Morris et al., 2013). Besides, mixotrophic protists can shift their nutrition type from autotrophic to heterotrophic following the availability of resources. They can strongly modify the microbial food web structure by modulating the relative importance of different energy pathways to higher trophic levels (Mitra et al., 2014). Competition for nutrients can also occur among phytoplankton species or between phytoplankton and bacteria (Bratbak and Thingstad, 1985). However, commensalism among phytoplankton and bacteria is not rare, especially at the end of the bloom when the availability of nutrients is low. This is because phytoplankton produces exudates, notably DOC, that benefit bacterial productivity (Bratbak and Thingstad, 1985; Gurung et al., 1999). This type of relationship can also become mutualistic when vitamins and macronutrients are produced by products from bacteria and support phytoplankton growth (Paerl et al., 2017; Seymour et al., 2017; Mayali, 2018). Mutualistic phytoplankton-bacteria interactions, along with competition for nutrients (Joint et al., 2002), strongly influence the microbial food web structure as they modulate the community composition and energy transfer (Rooney-Varga et al., 2005), and their effect can propagate to higher trophic levels. The community composition can also be modulated by other interactions, such as parasitism, which is considered a very common strategy in marine systems (Théodoridès, 1989; Skovgaard and Saiz, 2006). Some protists can be endo- or epibiotic parasites. So far, only a few parasites of planktonic hosts have been identified, and parasitism may be more important than reported (Skovgaard, 2014). Viral infection can represent a particular form of parasitism where the parasite is intracellular and uses the genetic tools of the hosts to develop. Viral lysis of both bacteria and phytoplankton has been described as ubiquitous in marine waters (Wommack and Colwell, 2000). It is considered as one of the principal causes of microbial mortality and contributes to the dissolved pool (Suttle, 1994, 2005).

During the past decade, several studies have tried to assess complexity and structure of the microbial food web through planktonic food web models (D’Alelio et al., 2016) including the analysis of association and correlation networks (Faust and Raes, 2012; Posch et al., 2015; Needham and Fuhrman, 2016). However, the modification of the microbial food web structure in the Mediterranean Sea through different periods of phytoplankton productivity still remains poorly documented, especially on a species level (D’Alelio et al., 2016; Zhou et al., 2018; Santi et al., 2019). With the present analysis, focus was made for the first time on inter- and intra-guild relationships, by implementing a systemic approach that combines data on species, taxonomic groups and size classes. Our expectation is that different microorganism associations prevail during such time frames and modify energy transfer in the microbial food webs. Furthermore, associations can also be very different between different years depending on the climatic conditions. Major stressors, such as water temperature increases, related or not to global warming, modulate the interactions between microorganisms and consequently modify energy transfer (Aberle et al., 2012). Water temperature increase benefits smaller phytoplankton cells (Peter and Sommer, 2012) and smaller heterotrophic flagellates, thus reducing the energy transfer efficiency to higher trophic levels (Moustaka-Gouni et al., 2016). Experimental studies showed that increase of water temperature directly impacts planktonic food webs as it alters the bottom–up/top–down balance in favor of top–down control (Kratina et al., 2012; Lewandowska et al., 2012; Shurin et al., 2012) and induces trophic cascades (Vidussi et al., 2011). Warmer waters also modify the metabolism of organisms by increasing microbial oxygen and carbon demand per unit production (Vázquez-Domínguez et al., 2007) and inducing higher microzooplankton grazing rates (Chen et al., 2012). Changes in the relative importance of functional traits, ecological interactions, and metabolic rates, which are caused by warmer temperatures, have the potential to remodel the structure of the microorganism networks following bloom and non-bloom periods and can consequently jeopardize the provision of energy to higher trophic levels (Aberle et al., 2012).

The comprehensive understanding of the microbial food web structure in marine waters, particularly in productive coastal areas is still lacking, making it difficult to predict its modification under the effect of different stressors such as future global warming conditions. Bloom events are crucial for these ecosystems as they provide a substantial part of the annual primary production and energy transfer supporting the food web (Carstensen et al., 2015). The objective of the present study was to identify the microorganism correlation networks in a shallow productive coastal area in order to suggest potential interactions between microorganisms. Results on microorganism interactions provide scenarios about the structure of the microbial food web during different periods of phytoplankton productivity and document modifications between two contrasted climatic years potentially related to the global warming. This work aims at investigating what are the differences in correlation network structure and key taxa by comparing (1) non-bloom and bloom periods in shallow coastal waters and by studying (2) how bloom and non-bloom are modified by climatic conditions of two contrasted climatic years in the same area. The microorganisms of the microbial food web of a Mediterranean coastal site (Thau Lagoon) were monitored. They encompass various groups, taxa or species (hereafter called groups/taxa/species), which include 1 virioplankton, 2 bacterioplankton (both Archaea and Bacteria), 46 phytoplankton, 4 heterotrophic flagellates, 28 naked ciliates, and 29 tintinnids. The study was carried out during two consecutive years, a typical Mediterranean climatic year (2015), and an exceptionally warm year (2016). The survey was performed during the spring in 2015 (two bloom periods) and from winter to spring in 2016 (one non-bloom and one bloom period). The winter of 2016 was the warmest on record, and it displayed abnormally high water temperature, absence of significant water cooling in winter, and slow temperature increase from winter to spring (Trombetta et al., 2019). It modified bloom phenology and composition, offering insights to understand potential changes affecting the bloom and non-bloom microbial network structure under global warming.

## Materials and Methods

### Study Site

The Thau Lagoon (Supplementary Figure S1) is a productive marine ecosystem located on the French coast of the northwestern Mediterranean Sea (43°24′00′′ N, 3°36′00′′ E). It is a shallow coastal lagoon of 75 km^2^ with a mean depth of 4 m and a maximum depth of 10 m (excluding deep depressions), which is connected to the sea by three channels. It is mesotrophic, with a mean turnover of 2% (50 days), phosphorus- and nitrogen-limited (Souchu et al., 2010), and characterized by large seasonal water temperature variations (i.e., from 4 in the winter to 30°C in the summer) throughout the year (Pernet et al., 2012). Apart from its ecological interest, it is a lagoon of economic relevance, mainly due to oyster farms representing 10% of the French production. Complementary data on hydrological parameters (water temperature, salinity, oxygen concentration, oxygen saturation and turbidity), meteorological parameters (air temperature, wind speed, wind direction, PAR, UVA and UVB) and corrected fluorescence of the chlorophyll *a* monitored at high frequency are available in open access in Mostajir et al. (2018) and Trombetta et al. (2019).

### Sampling Design and Planktonic Diversity and Abundance

To determine planktonic diversity and abundance, water samples were collected weekly at 1 m depth using a Niskin bottle. The sampling was carried out at a fixed station (Coastal Mediterranean Thau Lagoon Observatory: 43°24′53′′ N, 3°41′16′′ E) (Mostajir et al., 2018) near the Mediterranean platform for Marine Ecosystem Experimental Research (MEDIMEER) in Sète. The water depth at the sampling station is 2.5–3 m. The station is located at less than 50 m from the main channel connecting the lagoon to the sea, where the water residence is at its lowest (less than 20 days) (Fiandrino et al., 2012). Water samples were taken from January 8 to May 12, 2015 and from January 12 to June 14, 2016. For ease of reading, hereafter we define these two distinct periods as 2015 and 2016, even though summer and fall were not investigated. The following methods for estimating the abundance were applied with the goal of identifying also the less abundant taxa, but the underestimation of some rare taxa cannot be excluded.

The abundance of virioplankton was estimated by epifluorescence microscopy as detailed in Chen et al. (2001). For this procedure, 1.8 mL water samples were fixed with 0.02 μm filtered buffered alkaline formalin (2% final concentration) and stored at −80°C until analysis. Next, subsamples (0.3–0.4 mL) were filtered through 0.02 μm pore size Anodisc filters (Whatman). After staining with SYBR GOLD, the filters were air dried and mounted between a slide and glass cover slip with 30 μL of antifadent mounting medium (Citifluor). Virus-like particles were enumerated using an Olympus AX-70 epifluorescence microscope.

The abundance of small planktonic non-pigmented cells (including archaea, heterotrophic bacteria, and chemosynthetic bacteria, hereafter called bacteria), and naturally pigmented cells (small size phytoplankton < 6 μm) was identified by flow cytometry. For bacteria and phytoplankton (<6 μm), duplicate 1.8 mL subsamples were taken and fixed with glutaraldehyde following the protocol described in Marie et al. (2001) and then stored at −80°C until analysis. The abundance of High nucleic acid (HNA) and low nucleic acid (LNA) bacteria (Lebaron et al., 2001; Zubkov et al., 2001a,b), cyanobacteria < 1 μm, picoeukaryotes < 1 μm and 1–3 μm and nanoeukaryotes 3–6 μm was estimated using flow cytometry (FACSCalibur, Becton Dickinson) following the method described by Pecqueur et al. (2011). The sizes of bacteria, pico- and nanophytoplankton are approximate and were estimated in FCM analyses based on the position of fluorescent beads of 1, 2, and 6 μm on Side Scatter (SSC), which were added to all samples. Auto-fluorescing cells (phytoplankton) and SYBR-stained cells (bacteria) were analyzed in separated sub-samples.

The abundance, diversity and size of large size phytoplankton (6–200 μm) was estimated by microscopy as detailed in Trombetta et al. (2019). Duplicates of 100 mL subsamples were taken and fixed with 10 mL formalin solution (about 4% formaldehyde final concentration) and kept cold (4°C) until analysis. Subsamples (50 mL) were settled for 24 h in an Utermöhl chamber and phytoplankton cells were identified and counted under an inverted microscope (Olympus IX-70) until counting at least 200 cells of the dominant taxa. Non-abundant and rare taxa were not counted until 200 cells. Phytoplankton was identified to the lowest possible taxonomic level (i.e., species or genus) using phytoplankton taxonomic key (Sournia et al., 1986; Tomas, 1997).

To estimate the abundance of heterotrophic nanoflagellates (hereafter called HF), 30 mL aliquots were fixed with sterile filtered (0.2 μm pore size) formaldehyde (4% final concentration). Samples were preserved at 4°C in the dark until analysis. Subsamples (10 mL) were stained with 4′,6′-diamidino-2-phenyindole hydrochloride (DAPI) and filtered with 25 mm black nucleopore polycarbonate membranes (0.8 μm pore size). Filters were placed on a microscope slide and HF were enumerated with an epifluorescence microscope (Olympus AX-70) using UV illumination. Formaldehyde-fixed samples were analyzed at maximum 15 days after sampling. HF were grouped in four size classes of <3, 3–5, 5–10, and >10 μm (hereafter named HF1, HF2, HF3, and HF4, respectively) (Mostajir et al., 2015; Moustaka-Gouni et al., 2016).

To estimate the abundance, diversity and size of naked ciliates and tintinnids (Dolan et al., 2012), 125 mL of the samples were fixed with 2% Lugol’s iodine acid solution. Samples were preserved in a cold dark room (4°C) until analysis. Subsamples (100 mL) were settled in an Utermöhl chamber for 24 h and the cells were identified and counted under an inverted microscope (Olympus IX70) using ciliates taxonomic keys (Bérard-Therriault et al., 1999). Empty lorica were very rare (<1%) and not counted as a tintinnid.

### Microbial Network Construction and Analysis

Correlation networks were constructed using abundance data of all microbial groups/taxa/species (or nodes) encompassing 1 node for virus, 2 nodes for bacteria, 46 nodes for phytoplankton, 4 nodes for HF, 28 nodes for naked ciliates, and 29 nodes for tintinnids. First, 2015 and 2016 data sets were divided into bloom and non-bloom periods based on the Chl *a* fluorescence daily mean as an index of phytoplankton biomass as described by Trombetta et al. (2019). They defined blooms as periods (1) that started with at least two consecutive days of positive growth rates and (2) where the sum of the net growth rates over at least five consecutive days was positive. The end of the bloom was the day before 5 consecutive days with negative growth. There were three spring bloom periods in 2015. As the two blooms occurring in spring were consecutive and interrupted by only 2 weeks of negative growth, they were pooled into one data set to be compared with the spring bloom identified in 2016. The winter bloom and the non-blooms in 2015 were not included in the present network analysis. Correlation networks constructed merging data from non-consecutive periods of non-bloom during 2015 are in Supplementary Figure S4. In addition, there was one non-bloom period in 2016. Second, positive and negative correlations among groups/taxa/species were identified and used to represent correlation networks. Third, network descriptors were calculated for each network. For each period, namely bloom in 2015, bloom in 2016 and non-bloom in 2016, three networks were produced: one positive, one negative, and one combining positive and negative together. No networks were computed for the non-bloom periods of 2015 as they were non-consecutive and interrupted by several weeks of bloom. In total, 9 correlation networks were built.

Network nodes were defined using 1:1 correspondence with the microbial groups/taxa/species, and the network links between them were identified using the Spearman’s rank correlation method. This method was chosen as it has been previously described as the best approach for weekly or higher frequency abundance data of microbial communities, which allows the identification of non-linear relationships without leading to conspicuous inflation of significant associations (Posch et al., 2015). Auto-correlation of the time series was checked prior analysis (ACF; R package Forecast v8.10). A Monte-Carlo resample procedure was applied with 9999 iterations. Only correlations with estimated *p*-values < 0.05 were considered for network construction and analysis. The analysis focused on correlation networks illustrating microbial communities during bloom and non-bloom in 2016 and bloom period in 2015. Network links could either be negative or positive. Correlations can provide hypothesis for potential direct or indirect interactions among or between microoganisms. Therefore, in the present study negative correlations were suggested as parasitism, predation, and competition, whereas positive correlations were suggested as mutualism, commensalism, cross-feeding, or a similar response to resources or physical-chemical conditions (Faust and Raes, 2012; Fuhrman et al., 2015; Posch et al., 2015).

Various descriptors were used to characterize network structure and complexity. The numbers of groups/taxa/species (nodes, N) and the links between them (edges, E) indicate network size and total number of correlation relationships, respectively. The degree indicates the total number of significant correlations in which a node is involved. Mean number of edges per node (mean degree) and the degree distribution describe network complexity. Group/species/taxa with the higher degree are hubs and indicates key nodes for the structure and functioning of the community (Domin et al., 2018). The proportions of negative and positive correlations provide information on the relative importance of various potential interaction types (i.e., parasitism, predation and competition vs. mutualism, commensalism, and cross-feeding). Network analysis was first applied using full networks (combining of negative and positive correlations) and then separately on negative-only and positive-only networks.

To get more insights on the role of dominant group/taxa/species in the networks, constructed correlation networks were inspected by representing firstly biomass-dominant species, and secondly most connected species with other organisms (hub species). Biomass-dominant species were determined for each period (i.e., bloom 2015, bloom 2016 and non-bloom 2016) using mean biovolume of each group/taxa/species per mL calculated with the equivalent size diameter (ESD) and the abundance of the organisms. For phytoplankton, naked ciliates and tintinnids encompassing several groups/taxa/species, the dominant-biomass species were considered those representing 90% of the total biovolume per mL and were used to present dominant species networks. The most connected species (hub species) for each networks are the nodes with the higher degree.

### Statistical Analysis and Cluster of Group/Taxa/Species

Cell ESD was considered to cluster the group/taxa/species in ESD classes. Such clustering served to perform a comparative analysis between networks and was also useful for the investigation of differences in the abundance of groups between the years. First, HNA and LNA were pooled into the bacteria group while different size classes of HF were lumped into the HF group. Second, the mean ESDs of phytoplankton, naked ciliates, and tintinnids groups/taxa/species were identified using the literature and microscopy observations (Supplementary Table S1). For all the size classes, the groups/taxa/species were ordered according to their mean ESD and the Pruned Exact Linear Time (PELT) algorithm for optimal detection of changepoints was applied (Supplementary Figure S2) to identify size classes based on rupture points (Killick et al., 2012). PELT is an algorithm able to detect multiple rupture points through cost minimization functions over possible numbers and locations of changepoints, in our case in the organisms’ ESD distribution. It includes several established procedures for detecting changepoints, such as penalized likelihood and minimum description length. The PELT analysis was performed in R using the *changepoint* package [Killick et al. (2012) and detected: (1) four size classes for phytoplankton: <6, 6–12, 12–25, and >25 μm; (2) four size classes for naked ciliates: <20, 20–27, 27–50, and >50 μm; and (3) three size classes for tintinnids: <45, 45–80, and <80 μm].

Paired Wilcoxon signed-rank test was used to compare the mean abundance of the various microbial groups and size classes between 2015 and 2016 (e.g., phytoplankton < 20 μm in 2015 vs. phytoplankton < 20 μm in 2016). Sample dates were paired by week number (ISO 8601). For example, the sampling dates of January 8, 2015 and January 12, 2016 were paired as both correspond to the 2nd week of their respective year. As the sampling period in 2016 was 4 weeks longer than in 2015 (23 weeks against 19, respectively), the last four sampling dates were removed from the dataset for the comparison between mean abundances.

### Novel Approach to Detect the Statistical Differences Between Empirical Networks

A novel approach was introduced here to assess the statistical differences between empirical networks. Such approach is based on the use of random networks as null models and allows the indirect comparison between networks constructed using empirical data. For each network constructed using empirical data, 999 random networks with the same number of edges and nodes were assembled using the Erdõs-Rényi model (Erdõs and Rényi, 1960). The results extracted from the random networks were used for a procedure analogous to permutation analysis. The distribution of the number of edges between functional groups (e.g., virus, bacteria phytoplankton, HF, naked ciliates and tintinnids) or size classes in the 999 random networks was compared with the number of edges shared in the empirical networks constructed with abundance data. When the number of shared correlations in empirical networks was in the tails of distributions from random networks, the presence of a significant deviation was recorded. The thresholds for significance were set to 2.5 and 97.5% of distributions from random networks; values that lie either below the first or above the second limit corresponded to the numbers of shared links in empirical networks that were significantly lower or higher than in their random counterparts, respectively. If the number of edges shared between two functional groups in the empirical network was below the 2.5% threshold, the type of relationship was rare. If the number of edges in the empirical network was instead above the 97.5%, the type of relationship was dominant. Significant deviations from random models served first to detect the presence of non-trivial patterns. Where such significant deviations characterized one empirical network only (e.g., the 2015 network and not the 2016 network), differential responses in the comparison with random models were also interpreted as significant differences between the empirical networks.

## Results

### Microbial Community Phenology

**FIGURE 1:**
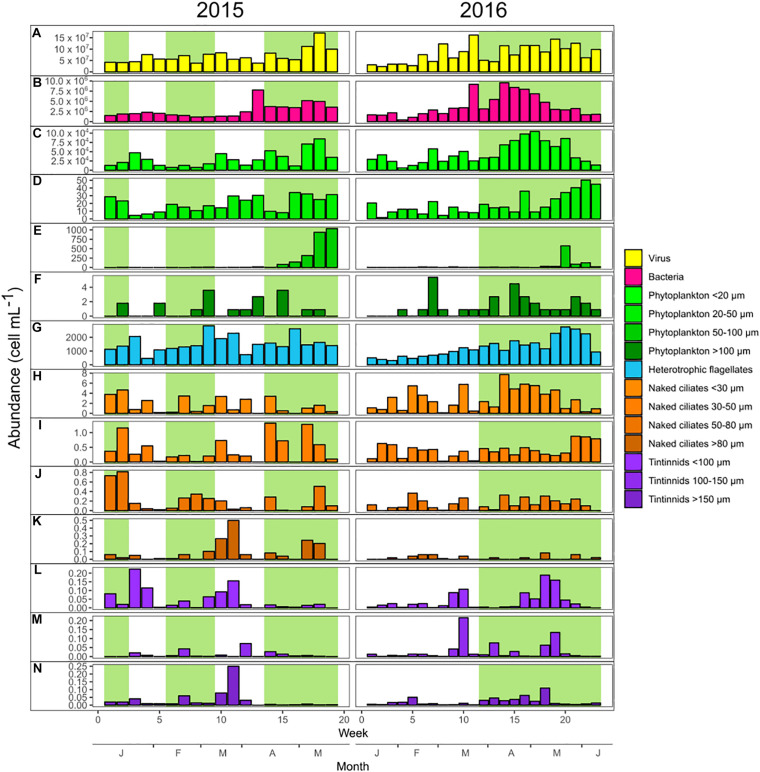
Weekly abundances of the main microbial plankton groups during 2015 (left panels) and 2016 (right panels). The bloom periods have green background and the non-bloom periods have white background. Yellow bars are for viruses **(A)**, pink for bacteria **(B)**, and light blue for heterotrophic nanoflagellates **(G)**. Bars of green gradient are for the different phytoplankton size classes (from **C–F**), orange gradient for naked ciliates (from **H–K**), and purple gradient for tintinnids (from **L–N**).

Abundance dynamic of planktonic groups, classified on the basis of size classes, are visualized in Figure 1 for the years 2015 and 2016. The results of the Paired Wilcoxon signed-rank tests presented in this section are detailed in Supplementary Table S2. The mean abundances of phytoplankton < 20 μm and naked ciliates < 30 μm were significantly higher in 2016 than in 2015 (*p-*values < 0.05). Bacterial abundances were also higher in 2016 but marginally significant (*p-*value = 0.06). Conversely, the abundances of phytoplankton 20–50 μm, HF, and naked ciliates > 80 μm were significantly lower in 2016 than in 2015 (*p-*values < 0.05). There was no significant difference between 2015 and 2016 (*p-*values > 0.05) in terms of the abundances of all the other groups including viruses, phytoplankton 50–100 and > 100 μm, naked ciliates 30–50, 50–80, and >80 μm and the three size classes of tintinnids.

### Correlation Networks of the Microbial Communities

#### Comparison Between 2015 and 2016 Bloom Networks

**FIGURE 2:**
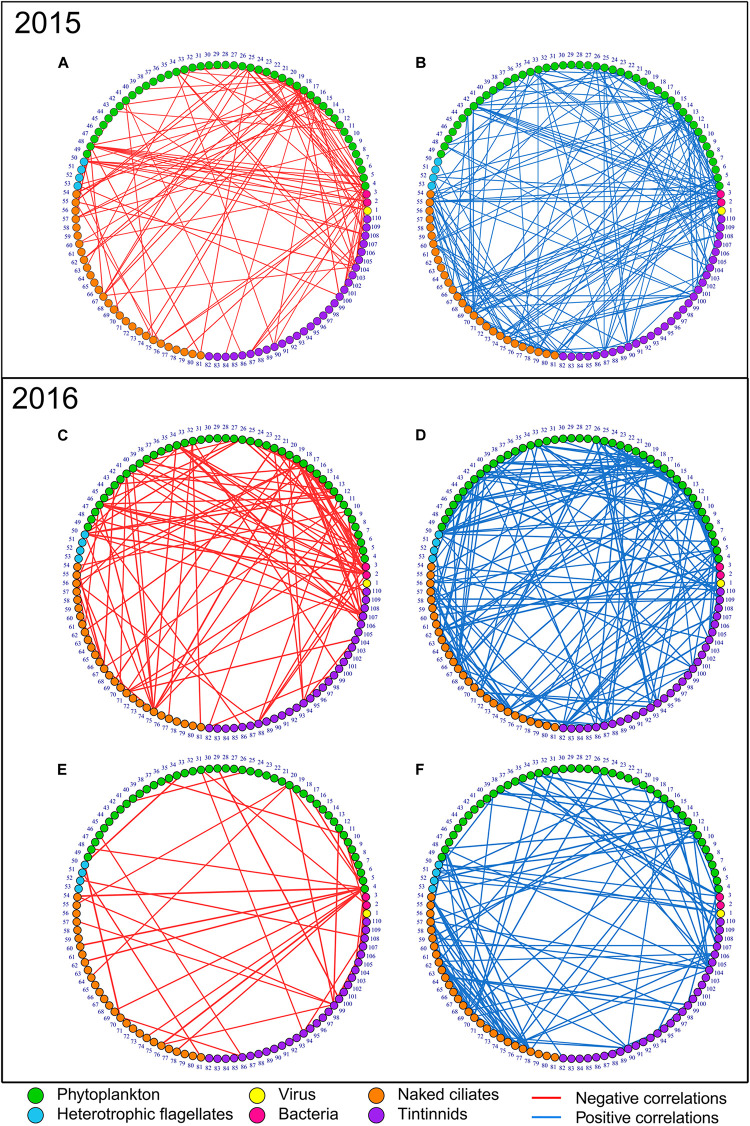
Negative (red edges, left networks) and positive (blue edges, right networks) correlation networks of the microbial communities in 2015 and 2016. **(A,B)** Present the spring bloom periods in 2015, **(C,D)** the spring bloom period in 2016, and, **(E,F)** the non-bloom period in 2016. Node numbers correspond to groups/taxa/species IDs as reported in Supplementary Table S1. Nodes are ordered by groups—viruses (number 1), bacteria (numbers 2 and 3), phytoplankton (from number 4 to 49), heterotrophic nanoflagellates (from number 50 to 53), naked ciliates (from number 54 to 81) and tintinnids (from number 82 to 110), and equivalent size diameter (ESD). Node colors: yellow, viruses; pink, bacteria; green, phytoplankton; light blue, heterotrophic nanoflagellates; orange, naked ciliates; and purple, tintinnids. The three networks combining both negative and positive correlations (full networks) are not presented in figures as they were obtained by simply merging the negative and positive correlations presented here. Consequently, only 6 networks out of the 9 produced are shown.

In Table 1 the descriptors of the various networks, shown in Figure 2, are presented. All combining negative and positive networks, negative networks, and positive networks of 2016 bloom have higher numbers of nodes (N) but a lower number of edges (E) than those in 2015 (Figure 2 and Table 1). The same percentage of negative edges was observed in 2016 than in 2015 (36%). Mean degree was slightly higher in 2015 than in 2016. In negative networks, the most connected nodes were *Lohmaniella* sp. (ID 67; 6 edges) for naked ciliates and *Tintinnidium* sp. (ID 104; 10 edges) for tintinnids in 2015, while it was *Leegardiella* sp. (ID 75; 9 edges) and *Eutintinnus rectus* (ID 107; 8 edges) in 2016.

**TABLE 1:** Network descriptors.

		Number of nodes (N)	Number of edges (E)	% Negative correlations	Mean degree
Combining Negative and Positive	Bloom 2015	67	295	36%	8.81
	Bloom 2016	79	288	36%	7.30
	Non-bloom 2016	62	153	27%	4.94
Negative	Bloom 2015	53	107		4.04
	Bloom 2016	59	103		3.50
	Non-bloom 2016	43	41		1.91
Positive	Bloom 2015	62	188		6.04
	Bloom 2016	74	185		5.00
	Non-bloom 2016	57	112		3.93

*The descriptors refer to the bloom periods for 2015 and 2016 and the non-bloom periods for 2015 and 2016, for the combining negative and positive, negative, and positive correlation networks. The descriptors are: the number of nodes (N), the number of edges (E), the percentage of negative correlations (for full networks) and the mean degree.*

#### Comparison Between Bloom and Non-bloom Networks of 2016

In 2016, the combining negative and positive networks, negative networks and positive networks (i.e., those including the full set of correlations) of the bloom period were more complex than those of non-bloom period as they presented higher N, E, and mean degree (Figure 2 and Table 1). The proportion of negative E during the bloom period was 9% higher in the bloom than in the non-bloom (36 and 27%, respectively). During the non-bloom period the most connected nodes were *Balanion* sp. (ID 54, 2 edges), *Cyrtostrombidium longisomum* (ID 70; 2 edges), and non-identified Holotriches. (ID 76; 2 edges) for naked ciliates and *Eutintinnus rectus* (ID 107; 7 edges) for tintinnids. Same results were observed for the non-bloom networks of 2015 (Supplementary Figure S4).

#### Correlation Networks of the Dominant Group/Taxa/Species

**FIGURE 3:**
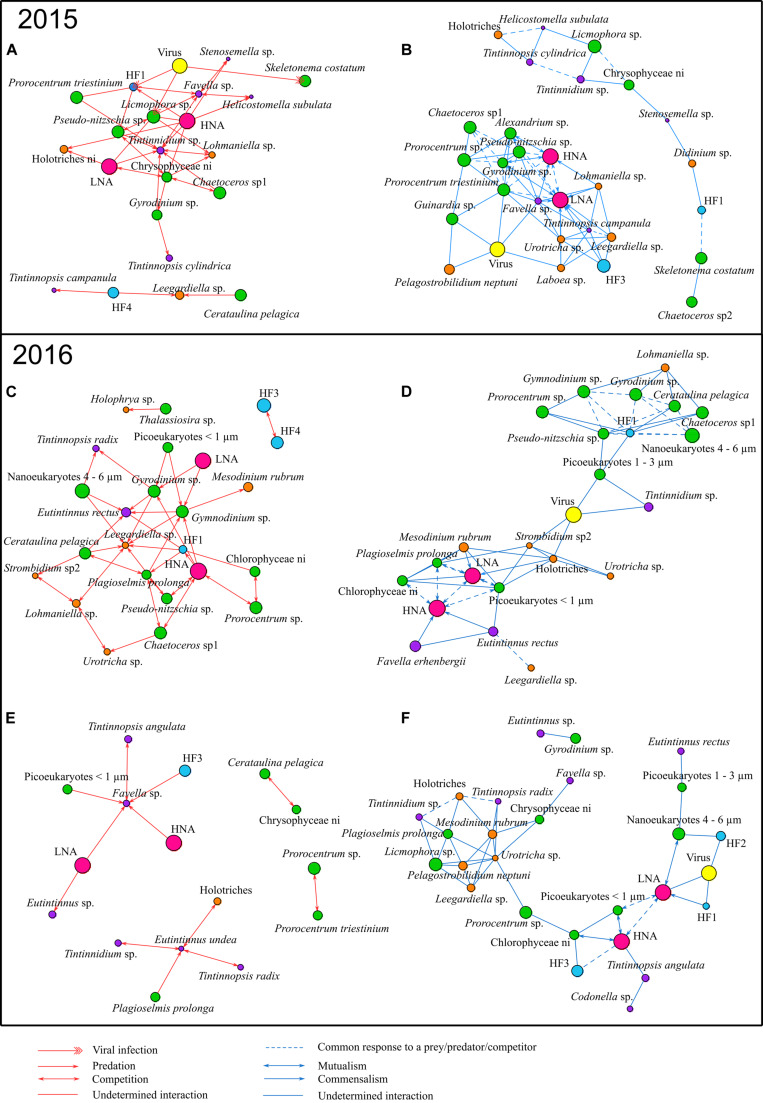
Correlations observed between dominant group/taxa/species in 2015 and 2016: **(A,B)** present the bloom period in 2015, **(C,D)** the bloom period in 2016, and **(E,F)** the non-bloom period in 2016. Red (left) and blue (right) edges correspond to negative and positive correlations, respectively. Node size is proportional to the mean biovolume over the period on a natural logarithm scale. Potential interactions for correlations are based on: (1) type of organisms involved, (2) their trophic function (predator, prey, etc.) in the food web, (3) their similar relationships with a common third organism (i.e., triangular structure) and (4) literature evidence. Red arrows represent potential predation, two-way red arrows potential competition, one-way double red arrows potential viral infection and red edges stand for undetermined negative interaction. Blue arrows represent potential commensalism, two-way blue arrows potential mutualism, dashed blue edges a potential common response to a prey/predator/competitor and blue edges undetermined positive interaction.

Significant correlations between the dominant group/taxa/species and identification of their potential interactions are presented in Figure 3. The abundance dynamics of these dominant group/taxa/species during the three studied network periods are shown in Supplementary Figure S3.

*Tintinnidium* sp. and *Lohmaniella* sp. were key ciliates species during the bloom of 2015 which showed the most important number of negative correlations with lower trophic levels groups/taxa/species such as bacteria (HNA and LNA) and phytoplankton (Figure 3A). These negative correlations that occurred between potential predators as ciliates, and potential preys, as phytoplankton and bacteria, can be interpreted as potential predation interactions. The same kind of negative correlations of highly connected nodes to potential preys, which can be interpreted as potential predation, was also present in the bloom of 2016 (Figure 3C); however, in this latter case it involved other ciliates species as *Eutintinnus rectus* and *Leegardiella* sp. Furthermore, in the non-bloom of 2016, other tintinnid species, *Favella* sp. and *Eutintinnus* sp., showed negative correlations to these potential preys, especially with HNA and LNA (Figure 3E). Potential predation interactions on bacteria were also common during the non-bloom of 2015 (Supplementary Figure S4). In addition, during the bloom periods of both 2015 and 2016 (Figures 2C, 3A), a large number of negative correlations between dominant phytoplankton groups/taxa/species were identified and potentially represent competition for the use of common resources. For example, during the bloom of 2016, *Plagioselmis prolonga* was negatively correlated to *Pseudo-nitzschia* sp., *Chaetoceros* sp1 and *Cerataulina pelagica* interpreted as potential competition interactions. Furthermore, high number of negative correlations between dinoflagellates like *Gymnodinium* sp. and *Gyrodinium* sp., that are potential mixotrophs or even can be heterotrophs, occurred with lower size groups/taxa/species as bacteria and small phytoplankton. This was particularly observed in the bloom period in 2016 (Figure 3C) and could be interpreted as potential predation.

Positive correlation between two groups/taxa/species that are involved with a third organism through negative association (i.e., the presence of a triangular structure of correlations) can indicate indirect interactions between the two taxa. Such indirect interaction is a consequence triggered by the direct relationship of both with the shared organism (e.g., with this latter directly interacting via predation or viral infection). As an example, in the blooms of 2015 and 2016 (Figures 2D, 3B), a large number of phytoplankton group/taxa/species along with bacteria groups (LNA and HNA) were positively connected between them but also negatively to a third organism notably a ciliate and represents a potential predator of both bacteria and phytoplankton nodes (e.g., *Favella* sp., *Tintinnidium* sp., etc.; Figures 3A,C). Consequently, these correlations might be interpreted as a common response of the preys to a shared predator. However, positive correlations between two groups/taxa/species can also be interpreted as potential direct positive relationships as for example in both 2015 and 2016 bloom networks (Figures 3B,D), where bacteria groups HNA and LNA were highly connected with phytoplankton, naked and tintinnids ciliates, which might indicate mutualism or commensalism.

### Dominant and Rare Potential Interactions Between Microbial Groups

**FIGURE 4:**
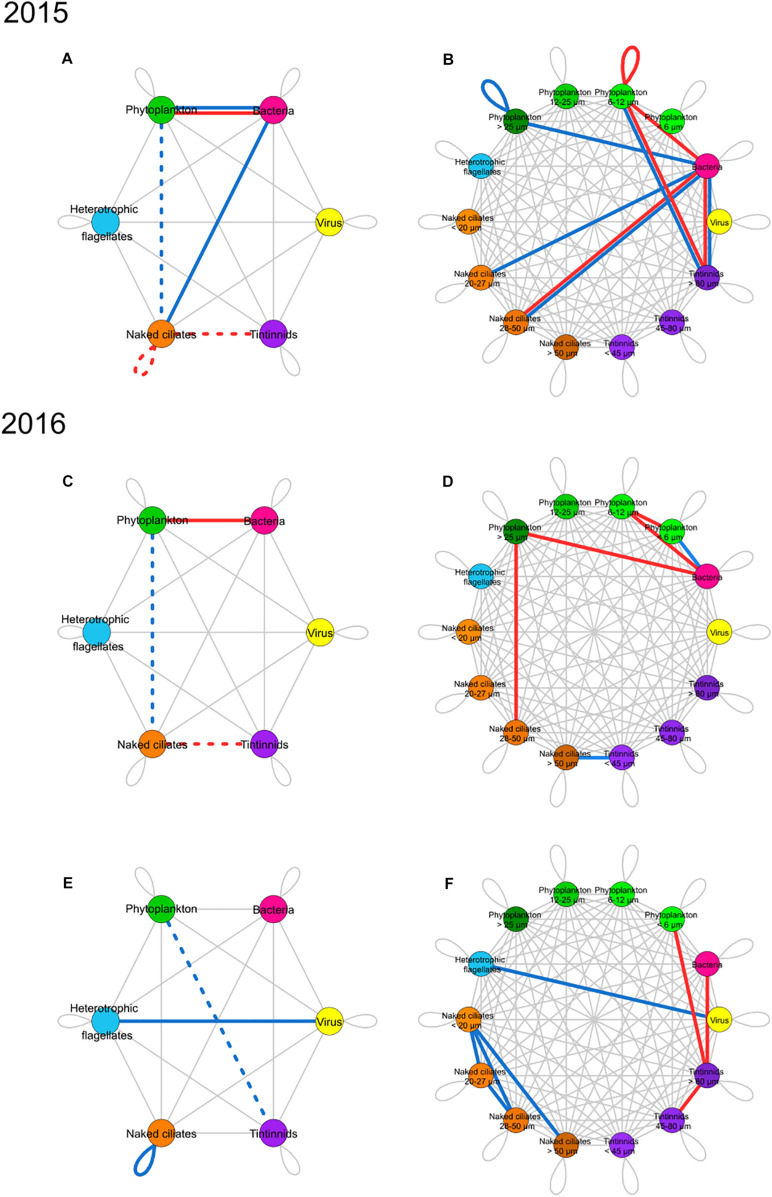
Summary networks of dominant and rare correlations between groups (left) and ESD classes (right) of microbial food web components. Rarity and dominance are defined according to the novel approach developed in the present study. **(A,B)** Present networks of bloom periods in 2015, **(C,D)** bloom period in 2016, and **(E,F)** non-bloom period in 2016. Left networks show correlations between groups while right networks show correlations between groups separated into ESD classes. Node colors: yellow, viruses; pink, bacteria; green, phytoplankton; light blue, heterotrophic nanoflagellates; orange, naked ciliates; and purple, tintinnids. Edges are colored when the number of correlations in networks constructed with empirical data significantly deviates from random models. Edge color provides information on the network type for which the significance was identified: (1) red edges correspond to negative correlation networks, (2) blue edges refer to positive correlation networks, (3) red and blue double edges are for deviations in both negative and positive correlation networks (i.e., deviations found in both networks, at the same time). Gray lines represent edges with numbers of correlations between groups that do not deviate from those in random networks. Edge type corresponds to the direction of the significance: (1) solid lines indicate more correlations than in random networks; (2) dotted lines stand for fewer correlations than in random networks.

Figures 4A,C,E shows which edges in the networks constructed from empirical data significantly deviated from random expectations between microbial groups; differences were tested by knowing the cumulative number of correlations portrayed by each edge between nodes. All the relationships described here are those that significantly deviated from null models. The bloom in 2015 was characterized by more negative relationships than random networks between bacteria and phytoplankton, and by more positive relationships between bacteria and phytoplankton, and between bacteria and naked ciliates (Figure 4A). This pattern did not occur in the bloom of 2016 (Figure 4B), as there were only more negative relationships between phytoplankton and bacteria. In 2015 and 2016 blooms, positive relationships between phytoplankton and naked ciliates and positive relationships between naked ciliates and tintinnids were less than those in random networks. In 2015 bloom, negative relationships within naked ciliates were also less than those in random networks.

During the bloom periods of both years (Figures 4C,D), the relationships between bacteria and phytoplankton and within the phytoplankton group prevailed, whereas the positive correlations between phytoplankton and naked ciliates and between phytoplankton and tintinnids were less than in random networks. During non-bloom in 2016 (Figure 4E), positive relationships prevailed between viruses and HF and within the naked ciliates group, and the correlations linking phytoplankton to tintinnids were underrepresented.

### Dominant and Rare Potential Interactions Between ESD Classes of Microbial Groups

Figures 4B,D,F summarizes what edges between ESD classes of microbial groups show numbers of correlations significantly different in empirical networks compared to random models. In the bloom of 2015 (Figure 4B), negative correlations prevailed between the groups at the bottom of the microbial food web, except the smaller phytoplankton ESD classes, and those at higher trophic levels. They involved phytoplankton 6–12 μm (self-loop) and occurred between bacteria and phytoplankton 6-20 μm, between bacteria and naked ciliates 28–50 μm, between bacteria and tintinnids > 80 μm, and between phytoplankton 6–12 μm and tintinnids > 80 μm. Positive correlations prevailed between bacteria and the largest ESD class of phytoplankton (i.e., >25 μm), between bacteria and naked ciliates 20–27 and 28–50 μm, between bacteria and tintinnids > 80 μm, and between phytoplankton 6–12 μm and tintinnids > 80 μm.

In the 2016 bloom period (Figure 4D), both negative and positive relationships prevailed among the groups at the bottom of the microbial food web, and in particular involved the smallest ESD classes (e.g., see the dominant positive relationships between bacteria and phytoplankton < 6 μm). Negative correlations that were overrepresented in empirical networks involved the relationships linking bacteria to phytoplankton 6–12 μm, bacteria to phytoplankton > 25 μm, phytoplankton < 6 μm to phytoplankton 6–12 μm and occurred between phytoplankton > 25 μm and naked ciliates 28–50 μm.

During the non-bloom period of 2016 (Figure 4F), negative relationships prevailed only between the smaller ESD class at the bottom of the microbial food web and the larger ESD class of tintinnids (i.e., between bacteria and tintinnids > 80 μm and between phytoplankton < 6 μm and tintinnids > 80 μm) Negative relationships also prevailed between tintinnids 45–80 μm and >80 μm. Positive relationships prevailed especially among naked ciliates ESD classes (i.e., between naked ciliates < 20 μm and 20–27 μm, 28–50 μm, and >50 μm, and between ciliates 20–27 μm and naked ciliates 28–50 μm).

## Discussion

In the present study, correlation networks were constructed to identify significant associations (i.e., both direct relationships and indirect relationships) linking microbial food web groups/taxa/species in coastal waters. An approach similar to those presented by Faust and Raes (2012) and Fuhrman et al. (2015) was applied in order to identify potential direct ecological interactions: negative correlations were interpreted as predation, parasitism, viral infection or competition, while positive correlations were considered as indicative of mutualism, commensalism or cross-feeding. Correlation network analysis was applied so far to a few plankton communities in marine and freshwater systems (Posch et al., 2015; Tan et al., 2015; Needham and Fuhrman, 2016; Xue et al., 2018). These studies had different objectives, such as identifying the daily succession of phytoplankton, bacteria, and archaea (Needham and Fuhrman, 2016), unraveling the inter-specific association involved in a bloom formation (Tan et al., 2015), describing the role of rare and abundant eukaryotic plankton in networks (Xue et al., 2018), or identifying phytoplankton-ciliates relationships during succession phases and comparing various correlation methods (Posch et al., 2015). Previous works investigated microbial food web interactions analyzing single networks representative of the bloom period while the present study aims at detecting differences between bloom and non-bloom periods of two distinct climatic years which one was warmer (2016) than the other (2015), therefore taking into account the consequences of this warming through the comparison of spring networks of these years. It focuses on the comparison between several networks resulting from the interactions among 110 groups/taxa/species of planktonic microorganisms, including virioplankton, bacterioplankton, phytoplankton, and protozooplankton grazers. It analyses the structure of microbial food webs components during bloom periods in 2015, a typical Mediterranean climatic year, and during bloom and non-bloom in 2016 which was characterized by an exceptionally warm winter. Weekly sampling was the optimal strategy taking into account the large number of groups/taxa/species and the time scale of blooms occurring in this particular site where bloom can occur during several months of blooms. Despite the rapid division rate of the organisms studied, weekly sampling allowed to highlight correlations between dominant groups/taxa/species. By combining the outcomes of the correlation analysis together with previous knowledge on the microbial food webs, we suggested which correlations most likely correspond to potential interactions. The reliability of our findings is supported by the fact that all putative direct interactions were identified among organisms that co-occurred for the major part of the studied periods (Supplementary Figure S3). Our application shows that network analysis is a powerful method to suggest hypotheses concerning the dominant processes and potential interactions that drive microbial food web dynamics. In the present work we identified: (1) most relevant microorganisms at the base of the microbial food web for energy transfer to higher trophic levels (e.g., phytoplankton-based vs. bacterioplankton-based food webs), (2) grazers that represent potential bottlenecks to energy circulation in the microbial food web and shifts in their relative importance during different periods (bloom versus non-bloom), and (3) key groups/taxa/species that are expected to modulate microbial network dynamics (i.e., hubs with larger numbers of connections compared to other nodes) in each period. By applying a novel approach to detect significant differences (Figure 4), which is based on the comparison between empirical and random networks, we found that there is a significant over-representation of both positive and negative correlations involving phytoplankton during bloom, which could potentially indicate increasing relevance of mutualism, grazing, and competition for resources (Figures 4A–D). Non-bloom networks were dominated by negative interactions between bacteria and tintinnids identified as potential predation of flexible omnivorous tintinnids on bacteria, and also positive correlations between ciliates groups, which suggests a shift of biomass transfer from phytoplankton-dominated food webs during bloom to a more bacterioplankton-based food webs during non-bloom (Figures 4E,F). However, correlations may refer to indirect relationships (e.g., they can involve taxa that lay several predator-prey interactions away from each other), and conclusions on the architecture of the main pathways for energy flow circulation should be carefully interpreted. Thus, suggested hypotheses based on these correlations require further measurements and future investigations. The warmer year favored the relationships among smaller organisms and increased the relevance of the microbial food web at the expense of the herbivorous food web.

### Network Complexity Increases During Phytoplankton Bloom

Microbial interaction networks during the bloom and non-bloom periods differ and complexity increases during phytoplankton blooms. Trombetta et al. (2019) showed that in the same shallow coastal waters, increase in water temperature during spring triggers phytoplankton bloom. Here, we found that increases in phytoplankton biomass are responsible for profound modifications at the level of microbial interactions. Such changes are particularly clear when comparing microbial networks during bloom and non-bloom periods in 2016. Bloom networks were more complex than non-bloom networks. They displayed higher numbers of nodes, edges, and mean degree than non-bloom networks, both when considering all correlations and in the presence of either positive or negative edges only. Such pattern indicates that there were generally more relationships among microbial organisms during phytoplankton bloom. Negative correlations between groups/taxa/species can stand for either predator-prey interactions or competitions (Faust and Raes, 2012). A hypothesis is that, during bloom, the increase in phytoplankton abundance supplied more resources to protozooplankton grazers, and the predator-prey interactions thus gained relevance. The increased importance of predator-prey interactions could also have been triggered by the high biodiversity of protozooplankton grazers, which enables longer pathways for biomass transfer from the bottom of the food web to the higher trophic levels (Moustaka-Gouni et al., 2016), as observed with potential paths of three steps during the 2016 bloom (i.e., Figure 3C, HNA – HF1 – *Leegardiella* sp.) For potential predator-prey interactions during the non-bloom period in 2016 only 6 negative correlations involved dominant group/taxa/species could have been highlighted; they include *Favella* sp., *Eutintinnus* sp. and *Eutintinnus undea* as predators (Figure 3E). In contrast, during the following 2016 bloom (Figure 3C) 17 potential correlations for predator-prey interactions were instead identified (excluding the interaction of the potential mixotrophic *Gyrodinium* sp. and *Gymnodinium* sp.), which suggests more efficient transfer to higher trophic levels due to a potentially larger number of pathways. *Lohmaniella* sp. (Supplementary Table S1, species number 67: ID 67) was the most connected node of naked ciliates in the negative networks during the 2015blooms (6 edges; Figure 2A). *Lohmaniella* sp. is a dominant naked ciliate in many marine ecosystems and mainly consumes phytoplankton (Chen and Chang, 1999). This is confirmed by the negative edges shared by *Lohmaniella* sp. with non-identified Chrysophyceae (ID 25), non-identified Cryptophyceae (ID 26) non-identified prymnesiophyceae (ID 18). All these taxa belong to the size class of small phytoplankton and comprised the favorable predation size range of *Lohmaniella* sp. (Kivi and Setälä, 1995; Tillmann, 2004). *Lohmaniella* sp. (ID 67) and *Tintinnidium* sp. (ID 104) during the bloom in 2015 and *Leegardiella* sp. (ID 75) and *Eutintinnus rectus* (ID 107) during the bloom in 2016 were both hub (most connected nodes) and biomass-dominant species (Figures 2A,C, 3A,C). These protozooplankton grazers are hubs (Domin et al., 2018) and can be seen as important key species that can modify microbial food web dynamics during phytoplankton bloom by consuming bacteria and small phytoplankton, and transferring relevant amounts of biomass to higher trophic levels. The present results also confirmed that HF are preferential grazers of bacteria (Figures 2A,C; LNA, ID 2 and HNA, ID 3), cyanobacteria (ID 4), and picophytoeukaryotes < 1 μm (ID 5), as shown previously by other investigations (Simek et al., 1997; Tophøj et al., 2018). The sharpening of competition could determine the complexity of the negative correlation network A relevant number of negative correlations among phytoplankton dominant group/taxa/species was in fact were observed in the blooms of 2015 and 2016 (Figures 3A,C, 4B,D). Such findings highlighted an important transition in the microbial network structure between the non-bloom and bloom period. Phytoplankton species compete for nutrients and light through physiological and biochemical adaptation (Margalef, 1978; Bratbak and Thingstad, 1985). Competition can manifest either indirectly through intrinsic physiological and biochemical adaptation, with the fittest species outcompeting the others (Krebs, 2001; Morin, 2009), or directly through allelopathy, mainly with species that produce chemical inhibitors (Rice, 2012). Models revealed that competition is an important factor that regulates planktonic community structure during blooms (Huisman et al., 1999; Hashioka et al., 2013; Sourisseau et al., 2017). Furthermore, field and laboratory studies identified competition among phytoplankton species during bloom (Sakshaug and Olsen, 1986; Sommer, 1988). However, this is the first time that *in situ* observations during bloom showed competition among phytoplankton groups/taxa/species as the potential predominant form of interaction shaping the microbial network. As an example, we found that the diatom *Chaetoceros* sp1 bloomed and was dominant during spring in both years, as it happens in many other systems during the same period (Cloern, 1996), including in the Thau Lagoon (Bec et al., 2005; Trombetta et al., 2019). This diatom was correlated negatively with other dominant group/taxa/species such as non-identified Chrysophyceae in 2015 or *Plagioselmis prolonga* in 2016, thus showing relationships that can be interpreted as competition.

The increase in complexity in the correlation networks during the blooms was also due to positive interactions. The higher number of positive interactions in the bloom compared to non-bloom period could have been the cause of the rising abundance of several phytoplankton groups/taxa/species. This might be explained by phytoplankton-produced exudates which benefit bacteria (Seymour et al., 2017), as there were more interactions between bacteria and phytoplankton compared to random expectations (Figure 4). Positive correlations between different groups/taxa/species of protozooplankton can also provide information about their potentially similar response to the phytoplankton resource. For instance, during the 2016 bloom, there was a positive correlation between *Eutintinnus* sp. and *Leegardiella* sp. These two species are known to be grazers of small phytoplankton (Kivi and Setälä, 1995; Gallegos et al., 1996) as it can be confirmed by negative correlations between them and nanoeukaryotes 4–6 μm The high abundance of nanoeukaryotes 4–6 μm (Supplementary Figure S3) during bloom allowed them to feed, develop, and coexist without competitive exclusion. The structure of the microbial food web between the bloom and the non-bloom periods was highly dynamic as shown by the presence of different key species in the two periods. Principal predators were different; for example, *Favella* sp., *Eutintinnus* sp. and *Eutintinnus undea* prevailed during non-bloom while *Leegardiella* sp., *Eutintinnus rectus*, and *Tintinnopsis radix* were dominant during bloom, which suggests a shift in the subset of species that link the bottom of the microbial food web to higher trophic levels. Changes in keys species dominance can be explained with the microbial succession related to bottom-up or top-down controls. Species can be controlled by (1) bottom-up forces such as physical-chemical parameters that directly affect physiology (e.g., temperature and salinity) or resources abundance (e.g., nutrients and prey availability) and (2) top-down forces such as predation or viral lysis. Both types of forces shape the community structure and interactions within the microbial food web (Mostajir et al., 2015). Furthermore, key species that engage in many potential predation interactions can indicate a more generalist trophic behavior (Faust and Raes, 2012; Díaz-Castelazo et al., 2013). The identification of key species based on the analysis of correlation networks can help pointing out groups/taxa/species that play central roles in energy transfer through the herbivorous food web and the microbial food web. Phytoplankton intraguild competition and interactions with bacteria dominate during bloom.

During 2015 and 2016 blooms, the number of interactions between bacteria and phytoplankton in the networks accounting for negative or positive relations, was significantly higher than what was found using random models (Figure 4). Interactions between bacteria and phytoplankton can indicate diverse relationships such as mutualism, commensalism, competition, or even predation in case of mixotrophic phytoplankton species (Danger et al., 2007; Mostajir et al., 2015). The high abundance and diversity attained by phytoplankton groups/taxa/species during bloom can explain the relevance gained by their associations with bacteria through increase in the available phycosphere (Seymour et al., 2017). First, bacteria can satisfy part of their own carbon demand by consuming phytoplankton exudates (Bratbak and Thingstad, 1985; Gurung et al., 1999). Second, positive correlations can indicate mutualism between bacteria and phytoplankton. Many phytoplankton species require several vitamins such as B_1_, B_7_, and B_12_ for their growth, which they are unable to synthesize (Seymour et al., 2017). Thus, vitamin-synthesizing bacteria provide vitamins to phytoplankton in exchange for organic carbon (Kazamia et al., 2012; Grant et al., 2014; Mayali, 2018). Moreover, it cannot be concluded that positive relationships between bacteria and phytoplankton simply indicate common response to a forcing factor such as the spring rising temperature, nutrients inputs, or predation (Fuhrman et al., 2015). Indeed, in the Mediterranean Sea, bacteria and phytoplankton community structure are known to be controlled by temperature (Bec et al., 2005; Zhou et al., 2018; Trombetta et al., 2019) influencing the microbial food web structure. Third, competition can occur between bacteria and small phytoplankton cells for nutrient resources, especially under limiting conditions (Joint et al., 2002). Bacteria may limit phytoplankton primary production by depriving it of nutrients.

### Potential Predation of Tintinnids on Bacteria Dominate During Non-bloom

During the non-bloom period of 2016, the number of negative correlations between bacteria and tintinnids > 80 μm was significantly higher compared to expectations from random models (Figure 4F). Such outcome, which may illustrate the prevalence of predation over bacteria, clearly deviates from the absence of dominant negative correlations between bacteria and potential predators during bloom (Figure 4D). In the network of dominant species during non-bloom (Figure 3), among the six negative correlations identified as potential predation interactions, three of them corresponded to those between bacteria (both HNA and LNA) and *Favella* sp. and *Eutintinnus* sp. (Figure 3E) which were already reported ingesting bacteria (Heinbokel, 1978; Turner and Tester, 1992). Moreover, during non-bloom of 2016 other (non-dominant) ciliates during the non-bloom period of 2016 such as *Pelagostrombilidium neptuni*, *laboea* sp., *Uronema* sp., *Balanion* sp., *Urotrichia* sp., *Mesodinium rubrum*, non-identified Holotriches, *Eutintinnus rectus*, *Tintinnopsis radix* and *Tintinnidium* sp. were negatively correlated with cyanobacteria (but not with bacteria). Therefore, correlation networks seem to indicate a shift from phytoplankton-based (autotrophic-based pathways) food web (Hlaili et al., 2014) during phytoplankton bloom to a more bacteria-based (heterotrophic-based pathways) food webs during non-bloom. In the 2016 bloom, the number of negative correlations between phytoplankton > 25 μm and naked ciliates 28–50 μm was in fact higher than in random models. No dominant negative correlations were instead found between bacteria and their potential predators (Figure 4D), suggesting the prevalence of predation over phytoplankton. In case of dominant species (Figure 3C), potential predation on phytoplankton was confirmed to prevail compared than potential consumption on bacteria. This shift is supported by 2015 data, with networks dominated by potential predation on bacteria during the non-bloom period (Supplementary Figures S4C,E), while during bloom the system was dominated by potential predation on phytoplankton (Figures 3A, 4B). Microzooplankton plays an important role in bacterial consumption and phytoplankton grazing, thus challenging the dominance of mesozooplankton in the herbivorous food web. Therefore, microzooplankton provides substantial amounts of energy to higher trophic levels through two complementary sources. The balance between the different sources can vary; our dataset shows that during the non-bloom periods, the phytoplankton biomass declined, thus impairing its capacity to sustain the growth of ciliates. Consequently, the tintinnids could have shifted their feeding toward bacteria. The shift to bacteria consumption allowed the maintenance of an efficient energy transfer from the bottom of the food web to higher trophic levels, as previously shown by Pomeroy and Wiebe (1988). Several studies have highlighted the relevance of bacterial production as an energy source in microbial food webs during pre- or post-bloom periods (Lignell et al., 1993; Christaki et al., 2014). Such shift in the trophic behavior of microorganisms between bloom and non-bloom modifying the food web structure and the transfer of energy have already been suggested through models, including for coastal waters of the Mediterranean Sea (D’Alelio et al., 2016), and the present study results corroborates this hypothesis. These shifts could also be the results of forcing occurring in coastal water, especially shallow ones. Shallow coastal waters including bays and lagoons are strongly influenced by physical and chemical forcing such as temperature, lands or marine inputs and benthic-pelagic fluxes (Rabalais et al., 2009). In the Thau Lagoon, water temperature is the predominant factor influencing the phytoplanktonic community structure (Trombetta et al., 2019) and shifts in trophic behavior of some tintinnids could have been due to the direct or indirect effect of water temperature.

At the end of phytoplankton bloom and during the non-bloom period when generally water temperature increase, positive correlations between the various groups of ciliates were dominant (Figure 4) and were identified among dominant group/taxa/species networks computed (Figure 3). These relationships could have been indirect relationship through a potential common response of ciliates to water temperature increase. Correlations among ciliates could also indicate a common response to copepods’ predation since this group is known to play an important role in controlling the dynamics of its preys (Calbet and Saiz, 2005).

### The Warmer Year Favored Relationships Among Smaller Organisms and Increased Potential Mixotrophic Predation

The characteristics observed in the 2016 spring bloom could depend on the exceptional warm winter preceding it. This is confirmed by the important number of nodes connected to water temperature, especially in 2016 (Figures 2, 3). Such an exceptional winter could have indeed caused the differences found when comparing the spring-bloom network structures of two consecutive years. During the bloom in the warmer year (2016), the relationships among small size groups/taxa/species were prevailing compared to those involving larger size organisms. The numbers of edges between bacteria and small phytoplankton < 6 μm, between bacteria and phytoplankton 6–12 μm, and between phytoplankton < 6 μm and phytoplankton 6–12 μm were higher compared to expectations from random networks (Figure 4D). The importance of the smallest size class of phytoplankton in the correlation network might be due to its higher abundance in 2016 than in 2015 (Figure 1 and Supplementary Table S2). During the 2016 bloom (Figure 3C), picoeukaryotes < 1 μm, nanoeukaryotes 4 – 6 μm and *Plagioselmis prolonga* were dominant and involved in various positive and negative correlations, including those with potential such as *Leegardiella* sp., *Eutintinnus rectus* or *Tintinnopsis radix.* That was not the case in the bloom of 2015 (Figure 3A). The dominance of small plankton in response to warming has already been described by *in situ* and experimental studies for both freshwater and marine systems (Peter and Sommer, 2012; Rasconi et al., 2015; Sommer et al., 2017). In microbial food web, warmer conditions promote the dominance of fast growing and r-trait species with small size and rapid development. In 2016, the positive correlations between viruses and smaller heterotrophic organisms (bacteria and HF) were overrepresented (Figures 4E,F). Virioplankton abundance is tightly coupled with the abundance of its hosts because the lytic viral cycle is fast (i.e., less than 24 h) and produces up to 500 viruses per cycle per host (for bacteriophages) (Wommack and Colwell, 2000). Thus, positive correlations could be interpreted as viral infections that follow the host abundance (Wommack and Colwell, 2000; Bettarel et al., 2003), especially with our weekly sampling. The predominance of correlations between virus and HF in 2016 could be explained by the probable positive effect of warmer conditions as temperature enhances viral infection and alters host susceptibility (Cottrell and Suttle, 1995; Nagasaki and Yamaguchi, 1998; Danovaro et al., 2011; Mojica et al., 2016).

During the bloom of 2016, the dinoflagellates *Gyrodinium* sp. and *Gymnodinium* sp. were both dominant and involved in a large number of negative correlations with smaller phytoplankton and bacteria (Figure 3C, i.e., LNA, HNA, picoeukaryotes < 1 μm, etc.), but not in the bloom of 2015. Dinoflagellates such as *Gyrodinium* sp. and *Gymnodinium* sp. are known to be mixotrophic and bacterivorous (Jeong et al., 2010). Thus, this kind of negative correlations could be interpreted as predation of mixotrophic organisms. Several studies reported that mixotrophic organisms, such as dinoflagellates, become more heterotrophic with rising temperature (Wilken et al., 2013). The present study shows that during the warmer year of 2016, potential mixotrophic dinoflagellates might have shifted their feeding mode to mostly heterotrophic nutrition. Therefore, with increased frequencies of exceptionally warm winters due to global warming in the Mediterranean Sea, the role of dinoflagellates in food webs could become more heterotrophic and contribute to the transfer of bacterial production to higher trophic levels. In the present study, the nutrition mode of *Gyrodinium* sp. and *Gymnodinium* sp. was not identified and no direct observations were made on feeding behavior. Therefore, these taxa could also strictly behave as heterotrophs.

A previous mesocosm study in the Thau Lagoon highlighted that warming increases primary production (Fouilland et al., 2013) leading to more efficient transfer of phytoplankton production to high trophic levels (Vidussi et al., 2011). However, due to the potential increase of relationships between smaller organisms this transfer could be less efficient than previously thought. The present study shows that during blooms in warm years there might be in fact an increase of ciliates and mixotrophic dinoflagellates predation on small phytoplankton. During warm years, the activity of protozooplankton predators could lead to the establishment of longer trophic chains and result in less efficient energy transfer from phytoplankton to higher trophic levels. The modification of microorganisms’ size during the bloom of warm years may affect various food web interactions up to the higher trophic levels (e.g., mesozooplankton, shellfish, and fish).

The effect of warmer conditions was highlighted comparing two contrasted climatic years that exhibited striking differences in terms of correlation network structure. These differences could have been also due to either direct (e.g., increasing metabolic rates, thermal tolerance) or indirect (e.g., predation rates modification) mechanisms linked to temperature. Moreover, various environmental factors can modulate interactions among microbial taxa. For instance, in shallow coastal waters, temperature and wind affect stability and mixing in the whole water column while sediment resuspension influences turbidity, nutrients, suspended organic materials and light availability. However, the fact that these differences between years were simply due to random variations cannot be excluded as already shown in other studies on correlation networks (e.g., see Figure 1 in Fuhrman et al., 2015).

### The Relevance of Trophic Cascades Intensified During the Warmer Year

The abundance of smaller microorganisms encompassing LNA and HNA bacteria, phytoplankton < 20 μm (mostly picophytoeukaryotes < 1 μm), and naked ciliates < 30 μm was higher in the warmer year (2016) than in 2015, whereas that of larger organisms including phytoplankton 20–50 μm (mostly diatoms and dinoflagellates), HF, and naked ciliates > 80 μm was lower (Figure 1 and Supplementary Table S2). Furthermore, smaller phytoplankton groups/taxa/species were dominant in 2016 involved in negative correlations with potential predators (Figure 3C; i.e., picoeukaryotes < 1 μm, nanoeukaryotes 4 – 6 μm and *Plagioselmis prolonga*); also longer potential food chains were identified (HNA – HF1 – *Leegardiella* sp.) but such pattern did not hold in 2015. These trends suggest that a trophic cascade could have reshaped the microbial community during the warmer year, leading to higher abundance of smaller microorganisms such as bacteria and small phytoplankton (<20 μm). The higher abundance of naked ciliates < 30 μm could have increased their grazing pressure above that of HF. Grazing may have reduced HF abundance, thus releasing various prey (i.e., bacteria and picophytoeukaryotes) from the top-down control of HF. The higher abundance of small naked ciliates (<30 μm) could be explained by the reduction of abundance and thus grazing pressure of metazooplankton, especially copepods and rotifers. However, as the present investigation focuses on microbial food web, copepods and rotifers were not adequately studied. The copepod-ciliate trophic link is a cornerstone relationship that transfers energy to higher trophic levels in several marine ecosystems (Calbet and Saiz, 2005). The reduction of copepods abundance during warmer winters has been previously reported in the literature (Mackas et al., 2006; Garzke et al., 2015). The same mechanism might be involved in this survey, with small naked ciliates showing higher abundance due to the release of top–down control from copepods. Models applied in mesocosms experiments revealed that changes in the balance between the various planktonic grazers and size classes strongly affect the top-down processes controlling the microbial food web structure (Larsen et al., 2015). The evidence that warming enhanced the trophic cascade in plankton communities has been presented both for marine and freshwater ecosystems (Vidussi et al., 2011; Kratina et al., 2012; Shurin et al., 2012). These studies suggested that warming shifted the microorganism community control from bottom–up to top–down.

The mechanisms underlying such shift are yet to be properly understood, but they may reflect different physiological responses between autotrophs and heterotrophs under warming conditions. Warm conditions seem to increase the metabolism of heterotrophic protists above that of autotrophs; thus, heterotrophs display more pronounced increase of growth and grazing rates in response to warming (Rose and Caron, 2007). These differential responses strengthen the trophic cascade as already reported by a *in situ* mesocosm experiment in the Thau Lagoon during spring (Vidussi et al., 2011). This trophic cascade strengthened benefit smaller microorganisms (Sommer and Lengfellner, 2008; Peter and Sommer, 2012). The temperature-size relationship can further explain why warmer conditions are more favorable for smaller organisms. In fact, increase in temperature makes smaller organisms more competitive than larger ones for the exploitation of resources, especially for CO_2_ and nutrient uptakes, due to the higher surface to volume ratio (Sommer et al., 2017). The volume-mediated response to warming may have played a significant role in the prevalence of smaller microorganisms and their interactions in the present study. Warming also allows predators to remain active during the winter and induces strong grazing pressure over the whole year (Sommer and Lengfellner, 2008).

## Conclusion

The present study unraveled that the structure of microbial correlation networks in Mediterranean coastal waters is highly dynamical and exhibits deep modifications following bloom and non-bloom periods. It highlighted characteristic correlations patterns between key species allowing to propose the potential hypothesis governing the microbial network structure. The abiotic environmental changes trigger low and high productive periods (i.e., non-bloom and bloom), thus shifting the dominance from bacterioplankton-based networks to phytoplankton-based dominated networks where predation on phytoplankton prevails, respectively. Moreover, inter-annual climatic variations deeply alter the arrangement of microbial potential interactions. Mechanisms under these changes could have been many, such as a shift from k to r-trait species, the enhancement of heterotrophic metabolism compared to autotrophic metabolism, the increase of growth and grazing rates, or difference in thermal tolerance. Our results provide a snapshot of what could occur in coastal waters of western Mediterranean Sea under prospective global warming, with exceptional warmer years becoming more frequent. Warmer waters enhance top–down control via the trophic cascade and support plankton communities where small-sized cells and microbial food web interactions dominate. There is a pressing need to understand how variations in the interaction network modify the structure of microbial communities in order to formulate reliable predictions about future global change scenarios. The analysis of correlation networks representing potential interactions among microbial groups/taxa/species in controlled experimental systems could help to elucidate the cause-effect mechanisms triggered by warmer temperatures (i.e., both constant increase and heat waves; see Pansch et al., 2018) and other factors related to climate change (e.g., ocean acidification and hypoxia).

## Data Availability Statement

All datasets generated for this study are included in the article/Supplementary Material.

## Author Contributions

FV and BM designed the experiment. FV, BM, and CR performed the experiment. FV, BM, MS, CR, and TT performed the analysis. TT wrote the original draft. All the authors contributed to manuscript revision as well as reading and approval of the final version.

## Conflict of Interest

The authors declare that the research was conducted in the absence of any commercial or financial relationships that could be construed as a potential conflict of interest.
