# Supplementary material for: Marine Microbial Food Web Networks During Phytoplankton Bloom and Non-bloom Periods: Warming Favors Smaller Organism Interactions and Intensifies Trophic Cascade
Source: Front Microbiol. 2020 Oct 23;11:502336. doi: 10.3389/fmicb.2020.502336 (PMC7644461; doi:10.3389/fmicb.2020.502336)
Supplement: Supplementary file 2 [file Data_Sheet_2.docx]

Supplementary Material

**Supplementary Figure 1.** Thau Lagoon and location of the sampling station. Grey background depicts lands. White background depicts water.

**Supplementary Figure 2:** Mean body size of groups/taxa/species for (A) phytoplankton, (B) naked ciliates, and (C) tintinnids. Red horizontal lines represent the ESD class identified using the PELT algorithm.

**Supplementary Figure 3:** Weekly abundance of the dominant groups/taxa/species that appeared in the networks of Figure 2. (A) Spring blooms of 2015; (B) Non-bloom of 2016 and (C) spring bloom of 2016.

**Supplementary Figure 4:** Networks of the 2015 non-bloom period. (A) Negative correlation network and (B) positive correlation network of the microbial communities. (C) Negative correlation network and (D) positive correlation networks of the dominant group/taxa/species. Summary networks of dominant and rare correlations (E) between groups and (F) between ESD size classes. For details on (A) and (B) see Figure 2 legend; For details on (C) and (D) see Figure 3 legend; For details on (E) and (F) see Figure 4 legend.

**Supplementary Table 1.** Groups/taxa/species used for the construction of the correlation networks. The following details are provided: group/taxa/species IDs, Equivalent Size Diameter (ESD), mean biovolume, taxonomic classification and ESD classes.

| Group/taxa/species | ID | Equivalent Size Diameter (ESD) (µm) | Biovolume (µm^3^) | Taxonomic classification | ESD Class |
| --- | --- | --- | --- | --- | --- |
| Virus | 1 | 0.2 | 0.004 | Virus | Virus |
| HNA | 2 | 0.8 | 0.268 | Bacteria | Bacteria |
| LNA | 3 | 0.8 | 0.268 | Bacteria | Bacteria |
| Cyanobacteria | 4 | 0.8 | 0.268 | Phytoplankton | < 6 µm |
| Picoeukaryotes < 1 µm | 5 | 0.8 | 0.268 | Phytoplankton | < 6 µm |
| Picoeukaryotes 1 – 3 µm | 6 | 2 | 4.19 | Phytoplankton | < 6 µm |
| *Fragilaria* sp1. | 7 | 4 | 31.42 | Phytoplankton | < 6 µm |
| *Fragilaria* sp2. | 8 | 4 | 31.42 | Phytoplankton | < 6 µm |
| *Pleurosigma* sp. | 9 | 4 | 47.12 | Phytoplankton | < 6 µm |
| Nanoeukaryotes 4 – 6 µm | 10 | 5 | 65.45 | Phytoplankton | < 6 µm |
| *Pyramimonas* sp. | 11 | 5 | 65.45 | Phytoplankton | < 6 µm |
| *Plagioselmis* *prolonga* | 12 | 5 | 78.54 | Phytoplankton | < 6 µm |
| *Chlorophyceae* ni. | 13 | 6 | 113.1 | Phytoplankton | 6-12 µm |
| *Dinobryon* *belgicae* | 14 | 6 | 130.9 | Phytoplankton | 6-12 µm |
| *Navicula* spp. | 15 | 7 | 212.06 | Phytoplankton | 6-12 µm |
| *Pseudo-nitzschia* sp. | 16 | 7 | 220 | Phytoplankton | 6-12 µm |
| *Leptocylindrus* *danicus* | 17 | 7 | 188.5 | Phytoplankton | 6-12 µm |
| *Prymnesiophyceae* ni. | 18 | 8 | 256.56 | Phytoplankton | 6-12 µm |
| *Heterocapsa* sp. | 19 | 8 | 251.33 | Phytoplankton | 6-12 µm |
| *Nitzchia* sp. | 20 | 8 | 261.8 | Phytoplankton | 6-12 µm |
| *Thalassionema* *nitzschioides* | 21 | 8 | 240 | Phytoplankton | 6-12 µm |
| *Chaetoceros* sp1. | 22 | 9 | 424.12 | Phytoplankton | 6-12 µm |
| *Chaetoceros* sp2. | 23 | 9 | 424.12 | Phytoplankton | 6-12 µm |
| *Diploneis* sp. | 24 | 9 | 353.43 | Phytoplankton | 6-12 µm |
| Chrysophyceae ni. | 25 | 10 | 523.6 | Phytoplankton | 6-12 µm |
| Cryptophyceae ni. | 26 | 10 | 523.6 | Phytoplankton | 6-12 µm |
| *Pseudopedinella* sp. | 27 | 10 | 523.6 | Phytoplankton | 6 12 µm |
| *Asterionellopsis* *glacialis* | 28 | 13 | 1309 | Phytoplankton | 1-25 µm |
| *Ceratoneis* *closterium* | 29 | 13 | 1309 | Phytoplankton | 12-25 µm |
| *Acanthoica* *quattrospina* | 30 | 14 | 1436.76 | Phytoplankton | 12-25 µm |
| *skeletonema* *costatum* | 31 | 14 | 1696.46 | Phytoplankton | 12-25 µm |
| *Thalassiosira* sp. | 32 | 14 | 1696.46 | Phytoplankton | 12-25 µm |
| *Eutreptiella* *braarudii* | 33 | 15 | 1832.6 | Phytoplankton | 12-25 µm |
| *Gyrodinium* sp. | 34 | 17 | 2945.24 | Phytoplankton | 12-25 µm |
| *Oxytoxum* sp. | 35 | 17 | 2945.24 | Phytoplankton | 12-25 µm |
| *Guinardia* sp. | 36 | 19 | 3926.99 | Phytoplankton | 12-25 µm |
| *Grammatophora* sp. | 37 | 20 | 4712.39 | Phytoplankton | 12-25 µm |
| *Rhizosolenia* sp. | 38 | 22 | 5890.49 | Phytoplankton | 12-25 µm |
| *Prorocentrum* sp. | 39 | 27 | 11453.72 | Phytoplankton | <25 µm |
| *Protoperidinium* spp. | 40 | 29 | 14137.17 | Phytoplankton | <25 µm |
| *Cerataulina* *pelagica* | 41 | 30 | 15707.96 | Phytoplankton | <25 µm |
| *Prorocentrum* *triestinium* | 42 | 30 | 16362.46 | Phytoplankton | <25 µm |
| *Alexandrium* sp. | 43 | 31 | 16493.36 | Phytoplankton | <25 µm |
| *Gonyaulax* sp. | 44 | 31 | 16493.36 | Phytoplankton | <25 µm |
| *Dynophysis* sp. | 45 | 34 | 22449.3 | Phytoplankton | <25 µm |
| *Gymnodinium* sp. | 46 | 36 | 28274.33 | Phytoplankton | <25 µm |
| *Scrippsiella* sp. | 47 | 36 | 28274.33 | Phytoplankton | <25 µm |
| *Licmophora* sp. | 48 | 40 | 37500 | Phytoplankton | <25 µm |
| *Euglenophyceae* ni. | 49 | 58 | 113097.34 | Phytoplankton | <25 µm |
| Nanoflagellates <3 µm | 50 | 2 | 4.19 | HF | HF |
| Nanoflagellates 3-4 µm | 51 | 3 | 22.45 | HF | HF |
| Nanoflagellates 5-10 µm | 52 | 7 | 220.89 | HF | HF |
| Nanoflagellates >10 µm | 53 | 12 | 904.78 | HF | HF |
| *Balanion* sp. | 54 | 9 | 392.7 | Naked ciliates | <20 µm |
| *Strombidium* sp1. | 55 | 9 | 445.06 | Naked ciliates | <20 µm |
| *Strombidium* sp2. | 56 | 9 | 445.06 | Naked ciliates | <20 µm |
| *Strombidium* sp3. | 57 | 9 | 445.06 | Naked ciliates | <20 µm |
| *Strombidium* sp4. | 58 | 9 | 445.06 | Naked ciliates | <20 µm |
| Scuticociliates | 59 | 11 | 670.21 | Naked ciliates | <20 µm |
| *Uronema* sp. | 60 | 12 | 1047.2 | Naked ciliates | <20 µm |
| *Strombidinopsis* sp. | 61 | 15 | 1769.76 | Naked ciliates | <20 µm |
| *Mesodinium* *rubrum* | 62 | 22 | 6370.63 | Naked ciliates | 20-27 µm |
| *Mesodinium* sp. | 63 | 22 | 6370.63 | Naked ciliates | 20-27 µm |
| *Strobilidium* sp2. | 64 | 22 | 6054.11 | Naked ciliates | 20-27 µm |
| *Strobilidium* sp3. | 65 | 22 | 6054.11 | Naked ciliates | 20-27 µm |
| *Tiarina* *fusus* | 66 | 23 | 6911.5 | Naked ciliates | 20-27 µm |
| *Lohmaniella* sp. | 67 | 24 | 8309.51 | Naked ciliates | 20-27 µm |
| *Coleps* sp. | 68 | 24 | 8377.58 | Naked ciliates | 20-27 µm |
| *Urotricha* sp. | 69 | 25 | 8869.76 | Naked ciliates | 20-27 µm |
| *Cyrtostrombidium* *longisomum* | 70 | 25 | 9047.79 | Naked ciliates | 20-27 µm |
| *Rhabdoaskenasia* sp. | 71 | 26 | 10305.99 | Naked ciliates | 20-27 µm |
| *Tontonia* sp. | 72 | 29 | 14137.17 | Naked ciliates | 28-50 µm |
| *Oligotriches* | 73 | 30 | 15079.64 | Naked ciliates | 28-50 µm |
| *Laboea* sp. | 74 | 33 | 20127.14 | Naked ciliates | 28-50 µm |
| *Leegardiella* sp. | 75 | 34 | 22449.3 | Naked ciliates | 28-50 µm |
| Holotriches | 76 | 34 | 23561.94 | Naked ciliates | 28-50 µm |
| *Holophrya* sp. | 77 | 51 | 78539.82 | Naked ciliates | >50 µm |
| *Pelagostrobilidium* *neptuni* | 78 | 62 | 143793.31 | Naked ciliates | >50 µm |
| *Didinium* sp. | 79 | 75 | 256563.4 | Naked ciliates | >50 µm |
| Euplotes | 80 | 75 | 256563.4 | Naked ciliates | >50 µm |
| Aloricate ciliates unidentified | 81 |  |  | Naked ciliates |  |
| *Tintinnopsis* *minuta* | 82 | 24 | 8181.23 | Tintinnids | <45 µm |
| *Acanthostomella* sp. | 83 | 26 | 10248.4 | Tintinnids | <45 µm |
| *Tintinnopsis* *koifoidi* | 84 | 29 | 14419.91 | Tintinnids | <45 µm |
| *Amphorides* sp. | 85 | 31 | 16755.16 | Tintinnids | <45 µm |
| *Tintinnopsis* *beroidea* | 86 | 36 | 28274.33 | Tintinnids | <45 µm |
| *Stenosemella* sp. | 87 | 37 | 28509.95 | Tintinnids | <45 µm |
| *Tintinnopsis* *corniger* | 88 | 39 | 36007.89 | Tintinnids | <45 µm |
| *Codonella* sp. | 89 | 42 | 44107.96 | Tintinnids | <45 µm |
| *Tintinnopsis* *baltica* | 90 | 42 | 44107.96 | Tintinnids | <45 µm |
| *Helicostomella* sp. | 91 | 42 | 44244.1 | Tintinnids | <45 µm |
| *Salpingella* sp. | 92 | 47 | 62831.85 | Tintinnids | 45-80 µm |
| *Tintinnopsis* *Lindeni* | 93 | 51 | 78848.74 | Tintinnids | 45-80 µm |
| *Tintinnopsis* *angulata* | 94 | 52 | 83168.43 | Tintinnids | 45-80 µm |
| *Tintinnopsis* sp. | 95 | 52 | 83383.11 | Tintinnids | 45-80 µm |
| *Tintinnopsis* sp1. | 96 | 52 | 83383.11 | Tintinnids | 45-80 µm |
| *Tintinnopsis* sp2. | 97 | 52 | 83383.11 | Tintinnids | 45-80 µm |
| *Stenosemella* *ventricosa* | 98 | 56 | 104719.76 | Tintinnids | 45-80 µm |
| *Eutintinnus* *undea* | 99 | 58 | 114511.05 | Tintinnids | 45-80 µm |
| *Tintinnopsis* *campanula* | 100 | 59 | 122522.11 | Tintinnids | 45-80 µm |
| *Helicostomella* *subulata* | 101 | 60 | 125663.71 | Tintinnids | 45-80 µm |
| *Codonellopsis* sp. | 102 | 67 | 176138.63 | Tintinnids | 45-80 µm |
| *Tintinnopsis* *cylindrica* | 103 | 67 | 180955.74 | Tintinnids | 45-80 µm |
| *Tintinnidium* sp. | 104 | 80 | 307876.08 | Tintinnids | >80 µm |
| *Eutintinnus* sp. | 105 | 85 | 361911.47 | Tintinnids | >80 µm |
| *Tintinnopsis* *radix* | 106 | 88 | 404820.39 | Tintinnids | >80 µm |
| *Eutintinnus* *rectus* | 107 | 98 | 558319.92 | Tintinnids | >80 µm |
| *Favella* sp. | 108 | 104 | 684898.61 | Tintinnids | >80 µm |
| *Favella* *erhenbergii* | 109 | 134 | 1477805.18 | Tintinnids | >80 µm |
| Tintinnina unidentified | 110 |  |  | Tintinnids |  |

**Supplementary Table 2.** Mean abundance during weekly samplings and results of the paired Wilcoxon tests on the weekly abundance of the different microbial groups and size classes (2015 vs. 2016). The term ‘Group’ indicates the various size classes of the different groups. The parameter V and the *p*-values refer to the tests performed. Asterisks stand for the significant tests at a 0.05 threshold. Details concerning abundance dynamics are provided in **Figure 1**.

| Group | Mean abundance in 2015 (cells mL^-1^) | Mean abundance in 2016 (cells mL^-1^) | *V* | *p-*value |
| --- | --- | --- | --- | --- |
| Viruses | 6.96 × 10^7^ | 7.66 × 10^7^ | 86 | 0.7381 |
| Bacteria | 2.77 × 10^6^ | 4.15 × 10^6^ | 48 | 0.0602 |
| Phytoplankton <20 µm | 3.04 × 10^4^ | 4.71 × 10^4^ | 33 | 0.0108 * |
| Phytoplankton 20-50 µm | 19.67 | 13.00 | 150 | 0.0258 * |
| Phytoplankton 50-100 µm | 137.23 | 11.40 | 92 | 0.4776 |
| Phytoplankton >100 µm | 0.95 | 1.28 | 25 | 0.4971 |
| Heterotrophic flagellates | 1.54 × 10^3^ | 1.05 ×10^3^ | 155 | 0.0141 * |
| Naked ciliates <30 µm | 1.65 | 3.22 | 45 | 0.0446 * |
| Naked ciliates 30-50 µm | 0.43 | 0.33 | 114 | 0.4653 |
| Naked ciliates 50-80 µm | 0.21 | 0.15 | 102 | 0.7983 |
| Naked ciliates >80 µm | 9.00 × 10^-2^ | 1.87 × 10^-2^ | 129 | 0.0138 * |
| Tintinnids <100 µm | 4.73 × 10^-2^ | 4.35 × 10^-2^ | 82 | 0.8961 |
| Tintinnids 100-150 µm | 1.11 × 10^-2^ | 3.42 × 10^-2^ | 63 | 0.2049 |
| Tintinnids >150 µm | 3.05 × 10^-2^ | 2.55 × 10^-2^ | 91 | 0.8906 |
|  |  |  |  |  |
