# Supplementary figures and images for: Marine Microbial Food Web Networks During Phytoplankton Bloom and Non-bloom Periods: Warming Favors Smaller Organism Interactions and Intensifies Trophic Cascade
Source: Front Microbiol. 2020 Oct 23;11:502336. doi: 10.3389/fmicb.2020.502336 (PMC7644461; doi:10.3389/fmicb.2020.502336)

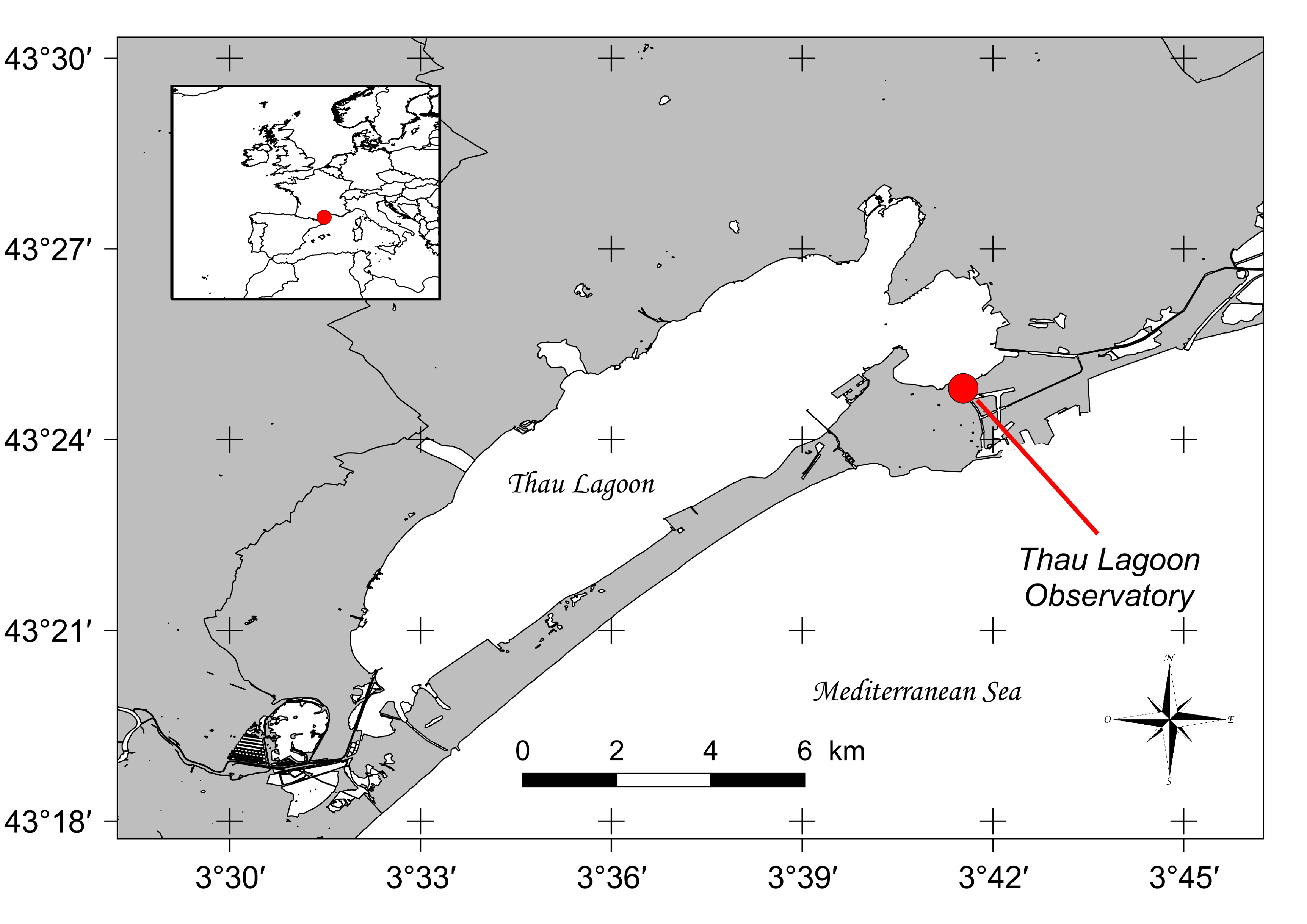

Supplement: Supplementary file 3 [file Image_1.TIFF]

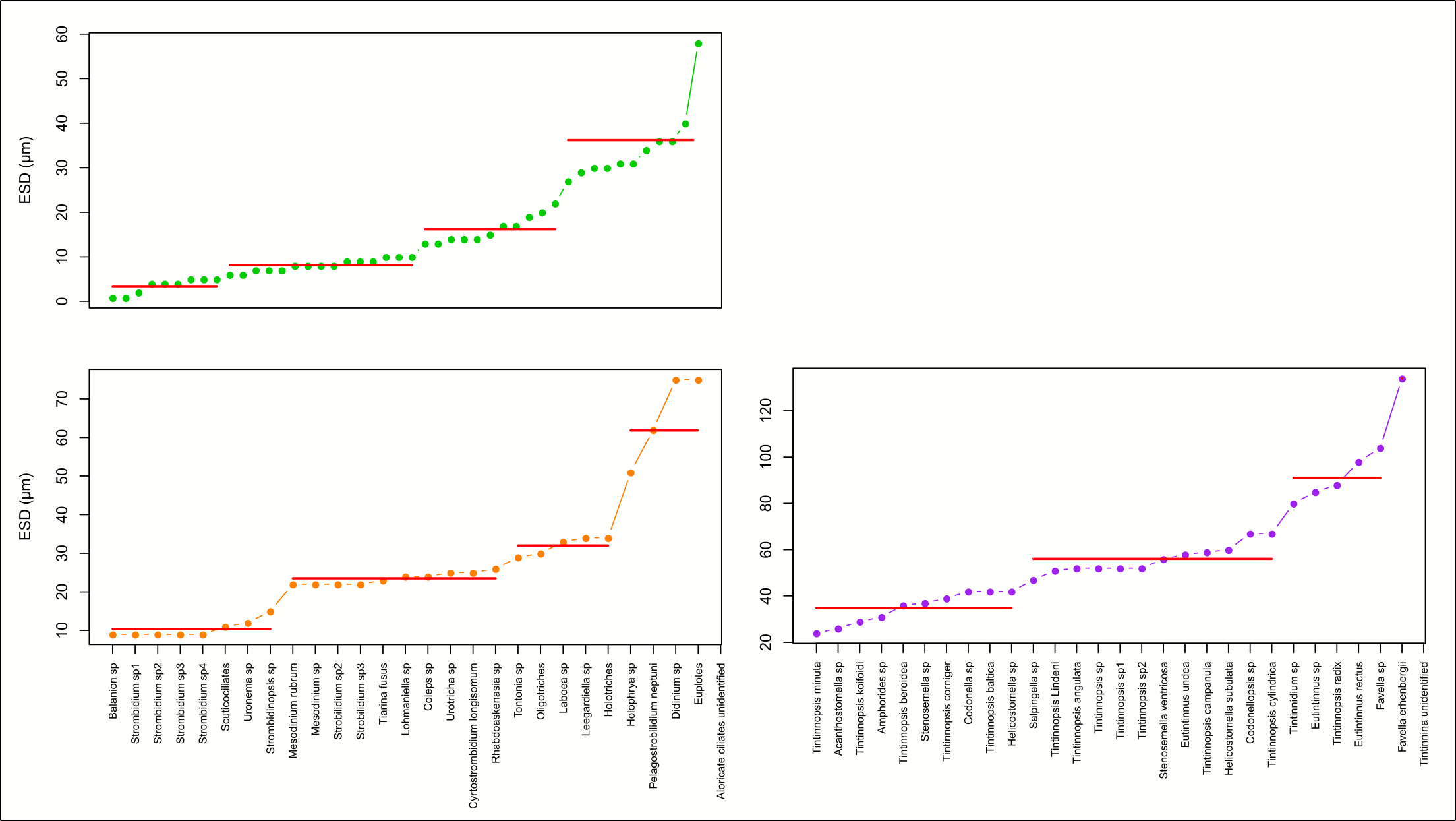

Supplement: Supplementary file 4 [file Image_2.TIFF]

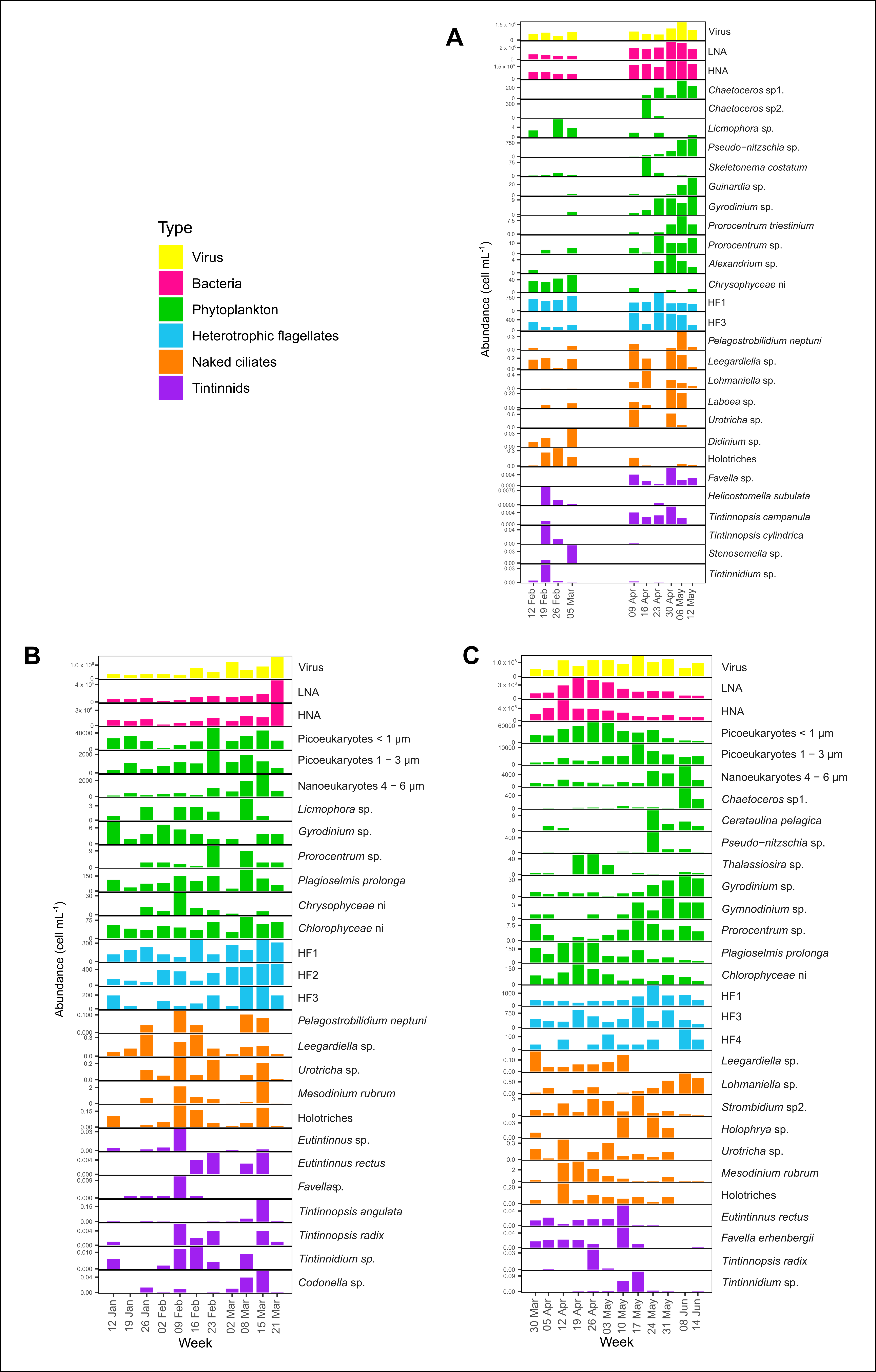

Supplement: Supplementary file 5 [file Image_3.TIFF]

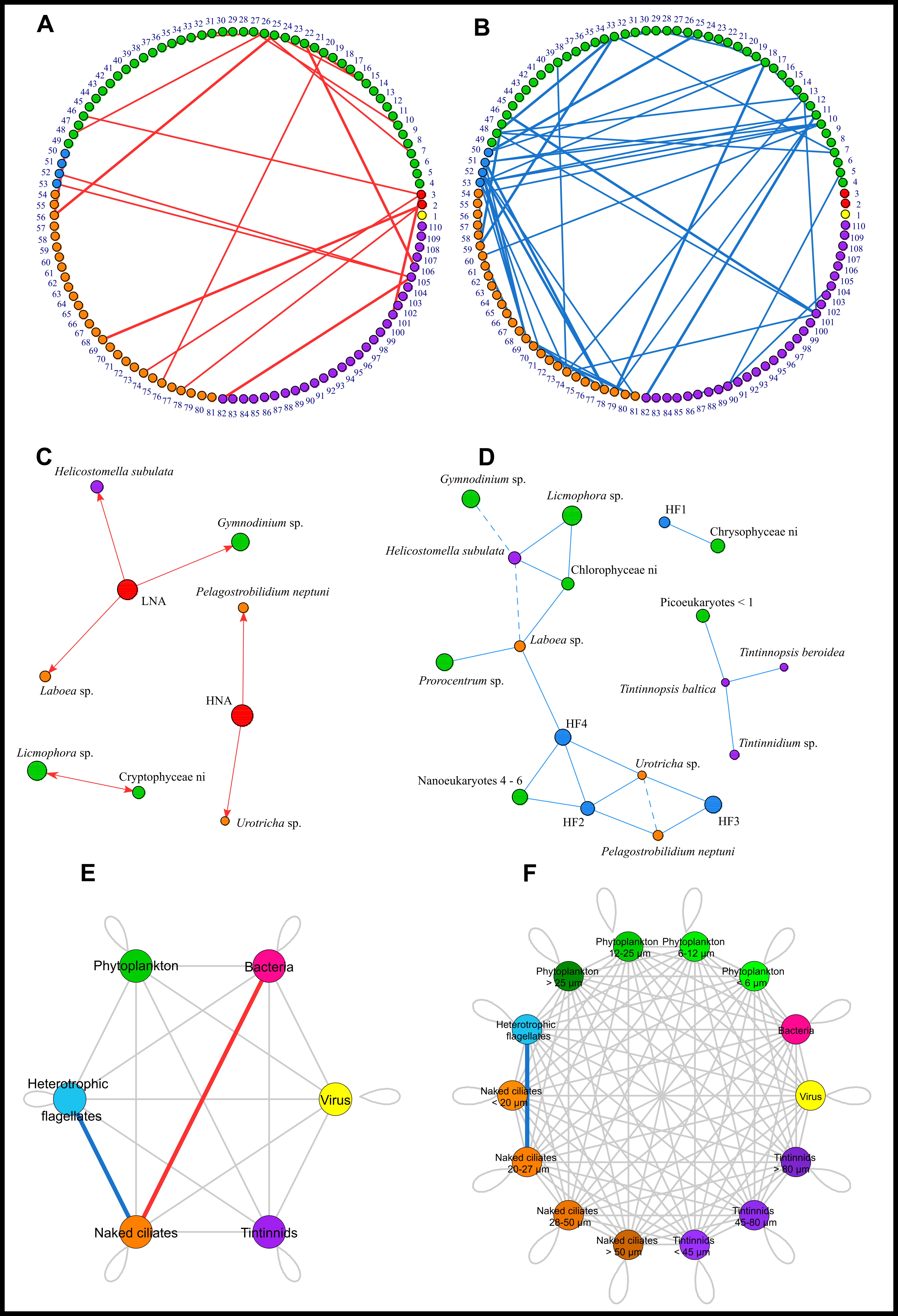

Supplement: Supplementary file 6 [file Image_4.TIFF]
